# Work-related injuries among farmers: a cross-sectional study from rural Nepal

**DOI:** 10.1186/s12995-016-0137-2

**Published:** 2016-10-26

**Authors:** Devendra Bhattarai, Suman Bahadur Singh, Dharanidhar Baral, Ram Bilakshan Sah, Shyam Sundar Budhathoki, Paras K. Pokharel

**Affiliations:** 1 School of Public Health and Community Medicine, B P Koirala Institute of Health Sciences, Ghopa 18, Dharan, Nepal; 2 Lifeline Institute of Health Sciences, Damak, Nepal

**Keywords:** Occupational Health, Farmers, Injuries, Rural Nepal

## Abstract

**Background:**

Agriculture work is one of the most hazardous occupations across countries of all income groups. In Nepal, 74 % of people are working in the agricultural sector. This study aims to identify patterns and factors associated with injuries among farmers of rural Nepal.

**Methods:**

A community-based cross-sectional study was conducted in a rural village in eastern Nepal. House to house visit was done to collect data from the farmers. The study included 500 farmers from Shanishchare village in Morang district of Nepal. A pre-tested semi-structured questionnaire was used to collect data on socioeconomic profile, agriculture work and injury. Prevalence of injuries among farmers in the last 12 months was calculated along with factors associated with the injuries.

**Results:**

The overall prevalence of work- related injuries among farmers was 69 % in the last 12 months. Common injuries among the farmers were cuts (79.7 %), puncture wound (11.3 %) and laceration (7.5 %). Hand tools were responsible for most of the injuries followed by slipping at work, sharp instruments, animals and fall from height. Upper limb injury comprised of 67 % of all injuries and the most involved part was fingers (43 %). The average number of years worked in farming by the respondents was 23.6 ± 13.6 years. Age and working experience of the farmers was found to be significantly associated with the occurrence of injuries among the farmers.

**Conclusions:**

The prevalence of injury among farmers in this study was high. Further research is needed to identify interventions to reduce the agricultural injuries in Nepal.

## Background

About 350,000 deaths occur globally due to fatal occupational injuries [1, 2]. The vulnerable population of South East Asian countries comprising of women, the poor, and children, are primarily employed in the informal sectors. They often lack the basic knowledge of hazards and work for long hours in unsafe work conditions without personal protection at work and with little or no health care insurance [3]. The use of machineries and equipment have led to newer occupational injuries among these workers [4, 5].

Agriculture has traditionally been one of the most hazardous occupations for workers [6, 7]. Agricultural sector provides a strong foundation for rural economic and for the sustainable economic growth [8]. An estimated 1.3 billion workers are engaged in agricultural production worldwide. This represents half of the total world labour force, and almost 60 % of them are in developing countries [9]. Agricultural injuries are reported from all around the globe [10–14].

In Nepal, agriculture contributes to 39 % of the gross domestic product with 13 % of the total foreign trade. Keeping in view of this contribution, priority is given to the development of the agriculture sector in the Eighth Five Year Plan [15]. In Nepal, 73.9 % of people are working in the agricultural sector and 26.1 % in non-agriculture [16]. The intensive use of machinery has raised the risks of injuries [17]. Musculoskeletal injuries are the predominant form of reported non-fatal occupational injuries. Fractures, bruises, lacerations, contusions, penetration by foreign bodies and sprains or strains are the most frequent type of occupational injuries [17–19].

### Occupational safety and health in Nepal

Approximately 20,000 work related accidents are estimated to occur every year and 200 lives are lost each year in Nepal due to work related injuries and accidents [20]. Occupational safety and health in Nepal is in its primitive stage [21]. Occupational safety and health has received limited attention by the health sector in Nepal [20, 22, 23]. The existing labour law has a small portion where the safety and health is a brief section with vague provisions for overall health and welfare of workers. The act has highlighted only four occupations; tea estate, construction, transportation and hotel & tourism sector separately. The law seems to focus only on increasing productivity rather than health and safety [21, 24]. Limited research is found in occupational safety and health and no research was found focusing on injuries among farmers [23]. Use of personal protective equipment (PPE) among farmers is not known and is reported low in other occupations [22].

Farmers are working under unsafe conditions, particularly in low income countries, leading to injury and death. Farmers are at risk for injury because agricultural work involves multiple tasks and multiple locations. Most of the tasks are carried out in the open air, exposing the workers to adverse working conditions. The majority of farmers are informal sector workers. Farming in villages is a household-based owned occupation and not a company owned business in Nepal. Literature searches in Pubmed, Google scholar and Nepal journal online resulted in the limited literature on injuries among farmers in Nepal. In Nepal there is no systematized recording and reporting of agricultural injuries. Data on injury at national level is also inadequate. This study was conducted to identify patterns of agricultural injuries and assess the factors associated with work-related injuries among agricultural farmer in rural Nepal.

## Methods

Morang district is the largest rice producer district of Nepal [25]. Shanishchare village was chosen randomly using the lottery method out of the 65 villages of Morang district. A community based cross-sectional study was carried out among farmers of the Shanishchare village in the eastern region of Nepal. This village is a highly populated village with a population of 29,804 and 5490 households, according to the population census of 2011. Based on the village data, agriculture work is the primary occupation of 19 % of the households in Shanishchare [26].

The sample size was calculated using the prevalence of work-related injuries among farmers, [10] in Hubei, People’s Republic of China. Taking prevalence of 33 % from this study and margin of error, as 15 % of prevalence, the sample size was calculated using the formula.

Sample size (*n*) = Z^2^pq/L^2^ *n*= (1.96^2^ *33*67)/4.35^2^ (where L= 15 % of p) {Z=1.96; *p*=33 %; q= compliment of p} *n*= 449.

Adding 10 % sample to correct non response, our expected sample size was decided as 494. Thus, we invited 500 farmers from the Shanishchare village to participate in this study.

Our Study period was from September 2012 to December 2013 which included the protocol designing, ethical approval, data collection and report preparation.

Farmers ≥ 20 years of age, having agricultural land ≥ 5 Katthas (1 Kattha = 3645 square feet), who worked in their own farm in Shanishchare village were included in our study. Farmers who are from Shanishchare village and working in fields outside Shanishchare village or farmers from other villages working on farms in Shanishchare village were excluded from our study. The reason for the exclusion of these farmers is that since farmers who work on other people’s land are more mobile and seasonal working for wages, or change occupation frequently, it is not feasible to include them in the study as well as disseminate the study findings afterwards. As per the Village Development Committee (VDC) office, there were 2500 farmers having land ≥5 Katthas for cultivation in Shanishchare. After the list of the 2500 farmers was obtained from VDC office, 500 farmers were selected using Systematic Random Sampling selecting every fifth farmer from the list. The first farmer was selected by generating a computer generated random number. However, some information bias cannot be avoided as the farmers were interviewed about injuries in the last 12 months, which is prone to some recall bias. We approached their home to conduct the interviews. If they were not present, we returned after arranging an appointment.

Socio demographic characteristics, work related data and injury characteristics were collected using a semi-structured questionnaire prepared by a team comprising of a Senior Public Health researcher, two Occupational Physicians, an Environmental health expert, a Biostatistician and a Master in Public Health student. The Semi-structured questionnaire was pretested among 50 farmers of Bayarban village, adjacent to Shanishchare village.

Working duration of the farmers was categorised taking 48 h as a cut off for working hours per week; and 20 years as cut off for years of working experience in this study. Both of these are based on the working duration criteria of the Labour Act of Nepal. The act states that the maximum number of working hours in a week should not exceed 48 h in occupation. It has provision of retirement from work after completion of 20 years in any occupation [24].

The collected data were checked thoroughly for completeness and entered in excel sheet after coding the data for analysis. Data Analysis was done using Statistical Package for Social Sciences (SPSS) version 11.5. Frequency and percentages are used to express descriptive statistics. Bivariate analysis of categorical data was done using χ^2^ test. Unadjusted Odds Ratio was calculated using Epi info 7. We calculated the 95 % confidence interval and the probability of significance was set at 5 %.

## Results

All 500 respondents approached for the study participated in this study giving a response rate of 100 %. The mean age of the respondents was 43.6 ± 13.2 years. There was an equal representation of male and female farmers in this study. All respondents in this study owned their own land for farming. The socio-demographic characteristics of the farmers in this study are shown in Table 1.

**Table 1:** Socio-demographic characteristics of farmers in Shanishchare village (*n* = 500)

Characteristics	Categories	Number	Percentage (%)
Age	<30 years	79	15.8
	30–39 years	121	24.2
	40–49 years	122	24.4
	50–59 years	107	21.4
	≥60 years	71	14.2
Gender	Male	246	49.2
	Female	254	50.8
Marital status	Single	57	11.4
	Married	443	88.6
Religion	Hindu	366	73.2
	Buddhist	25	5.0
	Kirant	97	19.4
	Christian	12	2.4
Literacy	Illiterate	86	17.2
	Literate	414	82.8
Types of family	Nuclear	242	48.4
	Joint	258	51.6
Land holding	≤15 Kattha	287	57.4
	>15 Kattha	213	42.6

The average number of years worked in farming by the respondents was 23.6 ± 3.6 years. More than 3/5th of the respondents, did not use any Personal Protective Equipment (PPE) at work. Among those who use PPE, 97.14 % use ordinary cotton mask, 4 % use boots and 1.7 % use gloves at work (Table 2).

**Table 2:** Working characteristics of the respondents (*n* = 500)

Characteristics	Categories	Number	Percentage (%)
Working hours	≤48 hours	119	42.2
	>48 hours	324	57.8
Work experience	≤20 years	183	36.6
	>20 years	317	63.4
Personal Protective Equipment (PPE) use	Yes	175	35.0
	No	325	65.0
Types of protective device (*n* = 175)^a^	Ordinary Mask	170	97.2
	Boot	7	4.0
	Gloves	3	1.7

^a^Multiple responses

A total of 345 respondents (69 %) reported being injured in the past one year. Among these 345 respondents, 9 out of 10 respondents were injured more than once in the past 12 months (Table 3).

**Table 3:** Injury related characteristics reported by respondents (*n* = 345)

Characteristics	Categories	Number	Percentage (%)
Environment where injured	Working field	314	91.0
	On the way	19	5.5
	House	12	3.5
Frequency of injuries (in past one year)	One time	37	10.7
	Two times	161	46.7
	Three times	121	35.1
	More than three times	26	7.6
Season when injured	Rainy season	221	64.1
	Winter season	124	35.9
Time when injured	Morning	107	31.0
	Day	209	60.6
	Evening	29	8.4

Hand tool was a frequent mode of injury among the respondents. Hand tools included sickle, axe, spade, hand saw and hoes. Most frequent types of injury were cut, and the site of injury was fingers (Table 4).

**Table 4:** Mode of injury, type of injuries and body parts injured (*n* = 345)

Characteristics	Categories	Frequency	Percentage (%)
Mode of injury	Hands tools	258	74.7
	Slipping	29	8.4
	Animals	27	7.8
	Sharp instruments	23	6.7
	Fall	8	2.3
Types of injuries	Cuts	275	79.7
	Punctures	39	11.3
	Laceration	26	7.5
	Fracture	5	1.4
Body parts injured^a^	Finger	151	43.8
	Leg	105	30.5
	Hand	70	20.3
	Head	23	7.9
	Knee	15	4.2
	Trunk	8	2.3
	Eye	5	1.4

^a^Multiple responses

A total of 222 (64.3 %) injured workers took some time off work due to injury. The mean (±SD) number of days lost due to injuries was 11.4 ± 9.6 days. Out of 345 injured respondents, 245 (71 %) of them used local herbs for first aid treatment. There were 233 (67.5 %) injured farmers who went to the health institution for wound treatment. Apart from herbs, human urine, mud, warm oil and toothpaste were used for first aid treatment of the injury. (Not shown in tables)

The association between socio-demographic characteristics and injuries among farmers is displayed in Table 5.

**Table 5:** Distribution of association between Socio- demographic characteristics of farmers by injuries in last one year in Shanishchare village

Characteristics	Accidents in last one year		*p*-value^a^	Unadjusted Odds Ratio	
	Yes (*n* = 345)	No (*n* = 155)		OR	95 % CI
Age in years					
<30	35	44	0.001	1	
30–39	86	35		3.08	1.70–5.58
40–49	86	36		3.00	1.66–5.41
50–59	87	20		5.46	2.83–16.56
≥60	51	20		3.20	1.62–6.33
Gender					
Male	162	84	0.134	1	
Female	183	71		1.33	0.91–1.95
Marital status					
Single	27	30	0.001	1	
Married	318	125		2.82	1.61–4.34
Literacy					
Literate	279	135	0.088	1	
Illiterate	66	20		1.59	0.92–2.74
Type of Family					
Nuclear	163	79	0.441	1	
Joint	182	76		1.16	0.79–1.69
Working hours ( per week)					
≤48 hours	127	84	0.001	1	
>48 hours	218	71		2.03	1.38–2.98
Working experience (years)					
≤20 years	159	98	0.001	1	
>20 years	186	57		2.01	1.36–2.96

^a^χ^2^ test

## Discussion

While there are only limited hospital or community based studies on injuries of all kinds in Nepal, no published literature was found regarding injuries among farmers in Nepal [27, 28]. This study could provide some evidences for further studies on agricultural injuries in Nepal. All the farmers owned their own land for farming in this study. However, it is a practice to work for another farm owner during need which is paid back by contributing equal number days in each other’s farm. This study showed that the majority of the farmers belonged to age group of 40–49 years, accounting for 24.4 % of all farmers. In this study, the mean age (±SD) was found to be 43.6 ± 13.2 years, which was similar to the findings of other studies [10–12, 29]. A cross-sectional study in India reports mean age (±SD) of farmers as 31.9 ± 6.6 years, which is much younger compared to our study [30]. Higher proportion of the injuries occurred among farmers in the age group 40–49 years. Another cross-sectional study in India shows high injury among farmers in the same age group as our study [31]. However, other studies show injuries among farmers in younger age groups [32]. This is explainable as the farmers in our study are comparatively older compared to the farmers in other studies. The overall prevalence of work- related injuries among farmers injury was 69 % in the last 12 months. Similarly, a cross-sectional study among agricultural workers in Ethiopia showed markedly high rates of injuries [11]. Both Nepal and Ethiopia are developing countries and are agrarian based and thus similar scenario can be seen. In Ethiopia, agriculture holds 41 % contribution to the gross domestic product, which is similar in Nepal, where agriculture contributes a similar proportion to the gross domestic product [16]. A study from India (30.6 %) shows a lower prevalence of agricultural injury compared to our study [33]. Incidence of injury among the farmers was lower in high income countries [34, 35]. The injuries among farmer are higher in low income countries compared to middle and high income countries. A case series study of surgical trauma and associated head injuries attending to a tertiary hospital in Nepal, reports one fifth of the injured patients were farmers [27]. A study of injury in an urban area of Nepal highlights that farmers suffered more injuries compared to workers in other occupations [28].

Out of 345 injured farmers, a large proportion of respondents (91.0 %) pointed farming fields as a spot for injury occurrence. Our study reported more frequent injuries compared to Ethiopia. Though Ethiopia is a low income country like Nepal, the use of machineries in farming and the techniques of farming may be different, which could explain the difference in findings [11].

One third of the farmers in this study report that they use personal protective equipment (PPE), however, almost all of these farmers only use ordinary masks at work, which they consider as PPE to be used in farming. This is a huge gap identified in our study. Use of PPE seems a neglected issue among farmers. They are not aware of any PPE required for farm work. Further researches may be needed to explore ways to increase access and the use of PPE by farmers.

Hand tools are the most frequent cause of injury in this study. The findings are similar in Indian farmers as well [30]. As a neighbouring country, the contexts of Nepal and India are comparable in population, culture, technology and practices. The findings are similar to the farmers from other countries [10, 11, 27, 36, 37]. Hand equipment like sickles and spades are still used routinely in the farms in Nepal. Cutting of grass, rice, wheat weeding, ridge formation, harvesting and irrigation channel making are done manually. This could explain hand tools as a major mode of injury in this study.

The most common types of injuries among farmers were cuts, puncture and laceration. Similar injuries were reported about Ethiopian farmers [11]. Popularity of traditional mechanical tools and not practicing safety measures could explain the prevalent injuries among the farmers.

Injuries were more common in hands than other parts of the body in this study similar to the study from Ethiopia [11]. Regular involvement of the fingers and hands in activities like cutting of grass and crops during working hours might increase risk of injury among them. Further, lack of safety precaution like use of personal protective equipment could put the farmers at more risk for injury.

There is similar proportion of males (49.2 %) and females (50.8 %) involved in agricultural activities. This highlights that women of Nepal are actively involved in agricultural activities besides regular household chores. National data of Nepal show slightly higher proportion (60 %) of women’s involvement in agriculture [20]. However, there is no significant difference in injuries among male and female farmers. Farm related injuries in Ethiopia showed that majority of study participants (77.8 %) were males [11]. Multiple studies report injuries among male farmers are more during farming [4, 38]. This could be explained as males are more involved in farming compared to females.

Comparable to the findings from Ethiopia, illiterate farmers were injured more than the literate farmers in this study [11, 15]. Further exploration may be needed as to why illiterate farmers have more injuries.

Farmers who worked for less or equal to 48 h a week, were less injured compared to those who worked for more than 48 h per week. Possible explanation could be that the farmers who work for more hours they will spend more hours exposed to the risk factors for injuries. The finding is similar in Ethiopian farmers [11].

There was a significant association between injury and the number of years worked as farmers. Similar findings were reported from Ethiopia [11]. This finding suggests that there may be a tendency of farmers to be less careful at work if they have worked for many years. This may also need further exploration.

Traditional practices are being practiced for first aid for injuries. This highlights the deep rooted traditional practice in our society. Local herbs are used for first aid and many do not visit a health institution at all. Injuries at work are perceived minor by these farmers. Traditional practices are also reported from India, where urination on wounds are practiced, as first aid [27]. Application of mud or cow dung on the injury site has been reported [33]. This shows that the farmers seem to lack skills and probably any knowledge about basic first aid.

### Limitations

The age and land ownership criteria for inclusion in this study may have left out daily wage seasonal agricultural labourers. The seasonal labourers are more migratory and they change working setup from agriculture to construction or other physical labour demanding works based on the availability of opportunities. Interviewing of only one farmer per land parcel may have left out the injury data on other workers in the family from the same farm. Only persons available at the time of study were included and we could not include the farmers who were not at home. There is possibility of recall bias in history of injury for last 12 months. The study could not identify a causal association for injuries at work among farmers. Other workers on the farm and the non-owners farm workers are not represented by this study. We have further plans to build on the findings of this research to conduct further research to address these farm workers.

## Conclusion

The most common types of injury among farmers were cuts, puncture and laceration. Most of the agricultural work is mechanical and farmers are found to be using traditional hand tools in Nepal. Laborious work, maximum use of hand tools, challenging work environment and neglecting safety measures could be responsible for occupational injury. While literatures are scanty in Nepal, this study provides evidence regarding injuries faced by farmers in Nepal, a country whose primary occupation is agriculture. Farming related stakeholders at village level, the agriculture administration at the local and national level, policy makers and researchers could use the findings of this study to design further studies to identify appropriate interventions to decrease injuries among farmers and address the occupational health needs of the farmers in Nepal.

## Abbreviations

PPE: Personal protective equipment
SPSS: Statistical Package for Social Sciences
VDC: Village Development Committee
